# Determinants of contraceptive use among Nigerian couples: evidence from the 2013 Demographic and Health Survey

**DOI:** 10.1186/s40834-017-0037-6

**Published:** 2017-01-17

**Authors:** Sarah R. Blackstone, Juliet Iwelunmor

**Affiliations:** Department of Kinesiology and Community Health, College of Applied Health Sciences, University of Illinois Urbana-Champaign, 123 Huff Hall, 1206S. 4th St., Champaign, IL 61802 USA

**Keywords:** Contraception, Couples, Male partner attitudes, Nigeria

## Abstract

**Background:**

Nigeria remains a focus for increasing contraceptive use, as it is one of the most populous countries in Sub-Saharan Africa. The objective of the current study was to investigate determinants of contraceptive use in Nigeria couples.

**Methods:**

Using the 2013 Nigeria Demographic and Health Survey, we estimated the likelihood of contraceptive use based on concordance with male partner desire for family size, male and female fertility preferences, female decision making power, and male partner attitudes toward contraceptive use.

**Results:**

Male partner perception that decisions regarding health should be made jointly or primarily by women was positively associated with use. Women were less likely to use contraceptives in couples in which male partners had greater earning power. Finally, men who viewed contraceptives as an enabler for promiscuity had female partners less likely to use contraceptives.

**Conclusions:**

These findings highlight the importance of male partners in women’s contraceptive decision making.

## Background

A large proportion of the population growth is occurring in the least developed parts of the world, particularly in Sub-Saharan Africa [1]. To address the population growth and the strain that it places on societal resources, there is an increased focus on strategies to reduce fertility rates, which has been incorporated in the Millennium Development Goals to improve maternal and child health. In many Sub-Saharan African countries, the goal of increasing contraceptive uptake remains tenuous [2]. Many programs throughout the area have received funding in an attempt to address the high levels of unmet needs in family planning. However, despite these programs, uptake of contraceptives in Africa has remained relatively slow [3].

Rates of maternal mortality are among the highest in the world in Sub-Saharan Africa, [4] sometimes as high as 1 maternal death per 100 births. Due to the low prevalence of contraceptive use, rates of unintended pregnancies are high, and as many as 50% result in elective abortions [5]. Abortions in Sub-Saharan African are often performed under unsafe and secretive conditions, with approximately 25% resulting in serious complications which account for 20–40% of maternal death [6, 7]. Family planning methods such as contraceptives can protect women from unintended pregnancies, thus reducing the number of unsafe pregnancies and abortions that make result. In fact, if high risk pregnancies were eliminated from Sub-Saharan Africa, maternal mortality rates could fall by 25% [4].

Many studies have identified misinformation, misperceptions, and fear of health side effects to be barriers to regular contraceptive use in Sub-Saharan Africa [8, 9]. Research also reports severe misconceptions about women’s own fertility and reproductive system. For instance, some female adolescents in Ghana did not believe they were old enough to get pregnant, though were at least 15 years of age [10]. Others believed that it was not possible to get pregnant during first intercourse. Also in Ghana, between 2003 and 2008, contraceptive use fell from 26 to 18% in large part due to fear of side effects from modern contraceptives. Hindin and colleagues [11] found that women felt that contraceptives caused illness and made them gain weight. Others were concerned that contraceptive use would cause a change in the menstrual patterns that could result in infertility later in life. In Nigeria, reasons for nonuse have included fear of side effects, partner objection, and religious conflicts, with the fear of side effects largely fueled by misinformation [5]. Evidence from other studies in Nigeria support that feared side effects also include damage the womb and difficulties with future ability to have children, [12] and in fact, women who are older and have had children are more likely to being accepting of, and use contraceptives [13].

The role of men in couples’ contraceptive choices cannot be ignored either. Because of the patriarchal society present in many Sub-Saharan African countries, men’s perceptions regarding contraceptive are the primary influences over couples’ behaviors [14, 15]. Studies have noted that women identify their fear of partner’s reaction or disclosure as a barrier to contraceptive uptake and use [14, 16, 17]. One study found that male partners’ disapproval for contraceptive use was as high as 84%, [18] and another study concluded that as many as 50% of women said that they would immediately discontinue use of a family planning method if their husband disapproved [19]. The societal importance of large families also poses an extreme challenge to regular uptake and use of modern contraceptives [8, 20].

Nigeria in particular remains a focus for increasing contraceptive use, as it is one of the most populous countries in Sub-Saharan Africa. Nigeria has a high total fertility rate (TFR), estimated to be between 5.5 and 5.7 for women of reproductive age (15–49). Low rates of contraceptive use are also pervasive in Nigeria [21]. Approximately 15% of married women report using contraceptives and 16% report an unmet need for family planning services [21]. The majority of contraceptive users in Nigeria rely on modern methods (10% of currently married women), 5% use traditional methods, 3% use injectable, and 2% use male condoms or pills as a method of contraception [21].

According to the most recent Demographic and Health Survey, rates of contraception in Nigeria have stagnated, remaining approximately 9% between 2008 and 2013. Though the contraceptive trend has not reversed, it is still concerning that contraceptive uptake is not increasing as it has been in other countries of Sub-Saharan Africa, thus underscoring the importance of research investigating factors that influence contraceptive use. In the current study we investigated determinants of contraceptive use at the couple’s level, with special attention to male attitudes towards contraception. Our study was guided by the following hypotheses:

1. Women in couples that show more male domination will be less likely to use contraceptives; also, we hypothesize that men’s desires (e.g. fertility preferences and desire for more children) will be more influential on women’s contraceptive use than their personal desires. This hypothesis was formulated based on the literature citing men as a dominating influence in reproductive health decision making in couples [14, 16–19].
2. Unfavorable male partner attitudes will be associated with decreased likelihood of using contraception. Here we seek to investigate the effects of men’s attitudes toward contraceptives on women’s contraceptive use within couples. This hypothesis is also based on the literature citing men, or fear of male partner’s reaction as a deterrent for contraceptive use [14, 16–18].

Better understanding factors that influence contraceptive use is necessary, as TFR is quite high and use of contraceptives remains suboptimal. The results of this study can assist with achieving the Millennium Development Goals of improving maternal and child health by determining characteristics that place couples more at risk for contraceptive non-use, and the role of male attitudes in these decisions [14, 22]. Though many efforts to increase contraceptive use are facing real challenges, few have taken into consideration the role of men influencing female partners’ decision making regarding contraceptive uptake [23]. As many reproductive health initiatives target individual women, considering the role of men in contraceptive behaviors is important since rates of contraceptive use are still suboptimal. This study builds on previous research [23, 24] to advance knowledge of the different roles that couples’ dynamics play in influencing the uptake and use of contraception in Nigeria.

## Methods

### Sample

We analyzed data from the 2013 Nigeria Demographic and Health Survey (NDHS). Many recent studies using NDHS data to investigate contraceptive determinants have examined the 2008 wave [25–28]. However, more recent studies that have used the 2013 wave have examined primarily individual determinants of contraceptive use, [29] and not determinants at interpersonal (e.g. couples) level. The NDHS sample was nationally representative and covered the entire population of non-institution dwelling residents. 40,000 households were included in the sample. The sampling design consisted of stratified three-stage cluster approach. Within each cluster, complete lists of households were generated, which resulted in a sampling frame for selection of households. Three questionnaires were used in the interview process: a household questionnaire, and separate men’s and women’s questionnaires. Women and men ages 15–49 were eligible to complete the surveys. A total of 38,948 women, 17,359 men, and 8658 couples completed the survey.

### Variables

#### Dependent variable

The primary outcome variable in this study was women’s contraceptive use. Women indicated whether they were using no method, a folkloric or tradition method, or a modern method. This was coded as follows (no method =0, folk/traditional = 1, modern = 2) as run as the dependent variable in a multinomial logistic regression.

#### Demographic information

Demographic information including age and education were adjusted for in the analysis. Age was self-reported in years and then categorized into 5-year age groups. Education was assessed by asking women to report the highest year of education they had attained. Women were asked to report the total number of children they had ever had, which was used as the measure of number of children in the current analysis. This measure did not specify whether all children were currently alive, or whether they were living with the mother. Women’s knowledge of contraception was accounted for by asking whether they were aware of various methods of contraception, and was recoded into a dummy variable assessing knowledge of at least one methods (1 = knows at least 1 method 0 = knows no method).

Demographic information was collected for men in the same format data were collected for women. Age was self-reported in years, and recoded into 5-year age groups. Education as assessed by asking men to report the highest year of education they had attained. Both men and women selected their region of the country (North central; North east; North west; South east; South central; South west), and their identified religion (Catholic; Other Christian; Islam; Traditionalist). As region of residence and religion showed minimal, non-significant effect sizes in preliminary analyses, they were not included as predictors in the final analysis. Men’s and women’s age and education were included in the final analyses.

#### Determinants of contraceptive use at the couple’s level

Both men and women’s desire for family size and fertility preferences were included in the analysis: men and women indicated whether they wanted more children, were undecided, or desired no more children. Women were then asked to report the concordance between their desire for family size and their husband’s, by indicating if their husband desired more children than the respondent, the same number of children, or fewer children than the respondent. Men were asked who should have more power in health care seeking decisions, with possible response being primarily men, joint decision, or primarily women. Women were asked who made majority of decisions regarding health care and allocation of funds within their family unit, with response options being primary men, joint decision, primarily women. Earning power within the couple was assessed by asking women to select from the following options: husband earns no money; woman earns more; both partners earn the same; male partner earns more.

#### Male partner attitudes

Male attitudes toward contraceptives were assessed using two indicators: 1) Contraception is a woman’s business and a man should not be involved, and 2) Using contraceptives make women promiscuous. Men were asked whether they agreed or disagreed with the previous two statements. There were transformed into dummy variables (1 = agree 0 = disagree), with those answering “do not know” removed from the analysis see Table 1 for full variable description.

**Table 1:** Description of variables

Variable	Abbreviation	Responses
Age in 5-year groups	Age/Men’s Age	15–19; 20–24; 25–29; 30–34; 35–39; 40–44; 45–49
Women’s contraceptive use	Contraceptive use	No method; Folkloric/Traditional; Modern
Women’s knowledge of contraception	Knowledge	Knows no method; knows at least one method
Education	Education/Men’s Education	Reported in years
Total children ever had	Total children	Self-report by women
Fertility preference	Fertility preference/Men’s fertility preference	More children; undecided; no more children
Husband’s desire for more children	Husband’s desire for children	More children than respondent; same number; less than respondent
Primary decision maker for health care	Health decision maker	Women; joint decision; male partner
Primary decision make for how money is spent	Spending decision maker	Women; joint decision; male partner
Men’s perception of who should have power in health care decision	Male perception of decision maker	Men; join decision; female partner
Earning power	Earning power	Husband earns no money; woman earns more; both partners earn the same; male partner earns more
Contraception is woman’s business and men should not be involved	Male attitude 1	Disagree; agree
Using contraceptives makes women promiscuous	Male attitude 2	Disagree; agree

### Statistical analysis

Using STATA 13, we estimated the probability of contraceptive use based on the three research questions detailed earlier. Multinomial logistic regression models were run to compute the logit and odds effects of individual determinants, couples’ determinants, and male attitudes on contraceptive use in women. The following two models were computed: 1) Probability of modern or traditional contraceptive use versus using no method, based on determinants in couples, and 2) Probability of contraceptive use based on male partners’ attitudes towards contraception. The following equation served as a reference for the two models discussed:

$$ \ln \left[\frac{ \Pr \left({Y}_i=1\right)}{ \Pr \left({Y}_i = 0\right)}\right] = {\beta}_0 + {\beta}_{1_{j\ {x}_1}} + {\beta}_{2_{j\ {x}_2}} $$

$$ \ln \left[\frac{ \Pr =\left({\mathrm{Y}}_{\mathrm{i}}=2\right)}{ \Pr =\left({\mathrm{Y}}_{\mathrm{i}}=0\right)}\right]={\beta}_0 + {\beta}_{1_{j\ {x}_1}}+{\beta}_{2_{j\ {x}_2}} $$

In this equation, the log of Pr (Y = 1) and Pr (Y = 2), respectively versus Pr (Y = 0), represents the probability of using folkloric/traditional methods versus no methods, and modern methods of contraceptives versus no method, respectively. Using this log transformation method in multinomial regression, the exponent of the regression constant (β0) is added to the regression coefficients of independent variables, where β1 is the effect age, β2 is the effects of male partner attitudes, and β3 is the effect of knowledge, etc. From this, the respective probabilities are calculated and the log of the proportion of the probability of Y = 1 versus Y = 0, and Y = 2 versus Y = 0 are computed. Log transformation allowed for the distribution of the categorical dependent variable to be normalized, facilitating interpretability.

## Results

### Descriptive statistics

14.5% of couples (1259) reported using any method of contraception. 4.63% of women used a folkloric/traditional method and 9.9% of women used a modern method. 22% of men agreed that contraception is a woman’s business that should not concern men, and 43.5% of men agreed that women who use contraception become promiscuous. On average, women attained 5 years of education (SD = 6.1) and men attained 6.9 years (SD = 5.8). The mean number of children per woman couple was 3.5 (SD = 2.5), with a maximum of 15 children. Women, on average were 28.5 years old (SD = 7.3). Men were an average age of 36.87 (SD = 7.03). Approximately 67% of couples resided in rural regions. See Table 2 for descriptive statistics.

**Table 2:** Descriptive statistics

Variable		N	Percent	Mean (SD)
Age	15–19	872	10.07	
	20–24	1674	19.33	
	25–29	2305	26.62	
	30–34	1703	19.66	
	35–39	1318	15.22	
	40–44	608	7.02	
	45–49	178	2.05	
Contraceptive Use	Yes	1259	14.5	
	No	7399	85.5	
Region	North Central	1412	16.31	
	North East	1719	19.85	
	North West	2851	32.95	
	South East	515	5.95	
	South Central	1043	12.05	
	South West	1118	12.91	
Urban/Rural	Urban	2821	32.58	
	Rural	5837	67.42	
Religion	Catholic	613	7.08	
	Other Christian	2808	32.43	
	Islam	5099	58.89	
	Traditionalist	80	.92	
	Other	3	.03	
Male Attitude (contraception is woman’s business)	Yes	1833	21.17	
	No	6463	74.65	
Male Attitude (contraception makes women promiscuous)	Yes	3608	43.5	
	No	4688	56.5	
Total number of children				3.0 (3.1)
Years of education				5.19 (6.13)

### Couple’s determinants

When we examined the effects of individual characteristics of men and women in couples, education levels for men and women were significant determinants of using folkloric methods (*B =* .037; *p <* .05; *B = .*055; *p <* .001) and modern methods (*B =* .059; *p <* .001; *B =* .057; *p <* .001) over no method. Men and women’s fertility preferences were determinants of folkloric method use (*B =* .298; *p <* .001; *B =* .372; *p <* .001), however only women’s fertility preferences predicted use of modern contraceptives (*B =* .577; *p <* .001). In couples in which the husband or male partner was the primary decision maker with regards to health care, women were less likely to use modern methods (*B = −*.252; *p <* .001) of contraception. Men’s perception that decisions regarding health should be made jointly or primary by women showed a positive effect on women’s use of folkloric methods versus no methods (*B =* .134; *p <* .05). Earning power showed a negative effect on use of folkloric methods (*B = −*.279; *p <* .05) compared to no method in couples with men earning more than women see Table 3. It should be noted that because numerical problems with the data, namely zero-cell count, knowledge was not run as a predictor of using traditional methods versus modern methods of contraceptive use. With regard to using any method of contraception versus using no method of contraception, knowledge was a significant predictor as expected (*B* = 1.24; *p <* .001) in the preliminary analysis.

**Table 3:** Couples’ determinants of contraceptive use in women

Folkloric/tradition vs. No Method		
	b/(se)	% Change in Odds
Age	−0.081 (0.08)	−7.8
Men’s Age	−0.054 (0.07)	−5.2
Education	0.055*** (0.01)	5.6
Men’s education	0.037* (0.02)	3.7
Fertility Preferences	0.372*** (0.09)	45
Men’s Fertility		
Preferences	0.298*** (0.09)	34.7
Total children	0.055 (0.04)	5.7
Husband’s desire		
For children	−0.028 (0.03)	−2.8
Health decision maker	−0.223**	−20
Spending decision		
Maker	−0.048 (0.06)	−4.7
Earning power	−0.279* (0.11)	−24.4
Male perception		
Of decision maker	0.134* (0.07)	14.4
Constant	−19.118 (877.29)	
Modern vs. No Method		
	b/(se)	% Change in odds
Age	0.009 (0.06)	0.9
Men’s Age	−0.006 (0.05)	−0.6
Education	0.052*** (0.01)	3.83
Men’s Education	0.059*** (0.01)	4.12
Fertility Preferences	0.577*** (0.07)	53.6
Men’s Fertility		
Preferences	0.115 (0.06)	12.0
Total children	0.036 (0.03)	3.7
Husband’s desire		
For children	−0.041* (0.02)	−4.1
Health decision maker	−0.252*** (0.05)	−22.3
Spending decision		
Maker	−0.070 (0.05)	−6.7
Earning power	−0.090 (0.09)	−8.6
Male perception		
Of decision maker	−0.018 (0.05)	−1.8
Constant	−19.720 (576.61)	

* *p* < 0.05, ** *p* < 0.01, *** *p* < 0.001

### Male attitudes towards contraception

Two indicators from the NDHS survey measured male partners’ attitudes toward contraception: 1) Contraception is a woman’s business and a man should not worry about it; 2) Women who use contraception become promiscuous. Neither measure of male attitudes significantly impacted women’s use of folkloric methods over no method. Men’s perception of contraception as a woman’s business also showed no associations with use of a modern method over no method. Men’s view that contraception makes women promiscuous was negatively associated with women’s use of modern contraceptives versus no method (*B = −*.284; *p <* .001). Both men’s and women’s education were significant determinants of using folkloric/traditional methods versus no methods (men: *B* =0.078; *p <* .001; women: *B* = 0.088; *p <* .001) and modern methods versus no methods (men: *B* = 0.087; *p <* .001; women: *B =* 0.084; *p <* .001) see Table 4.

**Table 4:** Effects of male attitudes on women’s contraceptive use

Folkloric/traditional vs. No Method		
	b/(se)	% change in odds
Male attitude 1	−0.085 (0.16)	−8.2
Male attitude 2	−0.001 (0.13)	−0.1
Age	0.080 (0.06)	8.4
Men’s age	−0.090 (0.06)	−8.6
Education	0.088*** (0.01)	9.2
Men’s education	0.078*** (0.01)	8.2
Total children	0.135*** (0.03)	14.5
Constant	−6.067*** (0.30)	
Modern vs. No Method		
	b/(se)	% change in odds
Male attitude 1	−0.090 (0.12)	−8.6
Male attitude 2	−0.284** (0.09)	−24.8
Age	0.182*** (0.05)	19.9
Men’s age	−0.074 (0.04)	−7.2
Education	0.084*** (0.01)	8.8
Men’s education	0.087*** (0.01)	9.0
Total children	0.128*** (0.02)	13.7
Constant	−5.192*** (0.22)	

* *p* < 0.05, ** *p* < 0.01, *** *p* < 0.001

## Discussion

This study highlights that decision making roles and power dynamics in couples are useful predictors of contraceptive use and that negative male attitudes towards contraception showed a negative relationship with using modern contraception versus no contraception, but showed no effect on women’s use of folkloric contraception versus no method. This finding underscores the importance of male partners for women’s use of contraception, both folkloric/traditional and modern methods. Both men and women’s fertility preference were important predictors of folkloric use; however it is noteworthy that only women’s fertility preferences were predictive of modern contraceptive use. One possible explanation for this could be that women using modern contraceptives are in more equitable relationships and have more control over their reproductive health [28, 30]. The finding that men as primary decisions for health care was negatively associated with modern contraceptive use, and men reporting that decision making regarding health should lie with women or be made jointly was positively associated with use of folkloric methods supports this notion. This is also further supported by the result showing that couples in which men earn more than women are less likely to having women that use contraception, which could possibly be a reflection of power dynamics in couples.

Our second finding suggests that fostering positive male partner attitudes towards contraception is important in increasing the use of modern contraceptives, which complements previous work such as Duze and Mohammed [18]. Surprisingly male partner attitudes did not affect the likelihood of women to use a folkloric method over no method. However, men’s belief that contraception is linked with promiscuity in women was negatively associated with use of modern contraceptives, which is consistent with previous literature regarding partner disapproval of contraception and women’s fear of men’s reactions and disclosure. It may be that regardless of partner’s attitudes, fear of partner reaction during disclosure may be so great that women are deterred from contraceptive uptake and use. Despite the lack of significant effects of male partner attitudes on folkloric/traditional method use, the negative effect of men’s perception of promiscuity among contraceptive users has implications for practice and policy. Working to decrease male partner stigma toward using modern contraceptive methods can be incorporated into future reproductive health initiatives, among other methods of targeting male partner knowledge, attitudes, and unmet need for contraception [17, 18, 31].

It is important to note that while both men and women’s education were significant predictors of folkloric/traditional method use and modern method use or not using contraceptives, the effect sizes and change in the odds of using contraceptives were minimal. The fact that these were significant predictors complements several previous studies identifying the importance of education as a predictor of contraceptive use [32]. However, the minimal effect size of education in these models accounting for couple’s determinants and male partner attitudes suggest that there are other relevant factors beyond individual’s education that have significant impacts on contraceptive use.

### Limitations

This study has a number of limitations. First, knowledge of contraception was dichotomous, so we were unable to determine the depth or accuracy of the knowledge women reported they had on contraceptives. Additionally, numerical problems with the data prohibited us from including knowledge as a covariate in the analysis. Second, the analysis would be strengthened by inclusion of more measures at the couple’s level beyond demographics, knowledge of and attitudes towards contraception. For instance, data measuring spousal communication regarding contraceptives, relationship trust, and marital satisfaction could be useful measures to investigate in future studies [33, 34]. Finally, there are limitations due to the study design. Because of the cross-sectional nature of this study, the results should be interpreted with caution as causality assumptions cannot be made. Furthermore, as the data were collected retrospectively there is a risk of recall bias, as well as other types of biases present in survey data collection including social desirability bias and response bias. Despite these limitations, these data provide a nationally representative sample of Nigerian couples to inform the literature on factors influencing contraceptive use.

## Conclusions

The findings support that the influence of men on contraceptive use indicates the need to include men in family planning indicatives [35]. Focusing on men’s attitudes towards contraception could improve communication and trust within couples, leading to less unintended pregnancies and reducing maternal and child mortality associated with unintended pregnancy. Furthermore, these results suggest the necessity of incorporating men’s unmet need for contraception and reproductive health programming in low-income, under-resourced countries. Overall, our results contribute to the current literature by providing an in-depth examination of modern, folkloric, and no contraceptive use in the context of couples’ characteristics. Specifically, we found that decision making roles and power dynamics in couples are important predictors of contraception, and actually differ between modern and folkloric use. Among couples in which male partners hold primary decision making power, women were less likely to use modern contraception. However, in more equitable relationships where decision making power was shared, or rested more heavily with the woman, folkloric methods were more common. Accounting for male partners and dynamics within couples as policies and practices move forward to address the issue of contraception is essential, as focusing solely on individual women is not sufficient to increase contraceptive uptake and use. Though larger cultural and social norms cannot be ignored, accounting for the significance of male partners in spousal contraceptive decisions can enable current and future reproductive health programs to work towards addressing unmet needs for contraception. This will be necessary as we move forward to address the Millennium Development Goals of improving maternal and child health by reducing unintended pregnancies, elective abortions, and subsequently maternal and child mortality.
